# Clinicopathological and prognostic significance of CXCR4 expression in osteosarcoma: a meta-analysis

**DOI:** 10.37796/2211-8039.1360

**Published:** 2022-12-01

**Authors:** I Gusti Ngurah Ananda Wira Kusuma, Grace Yulia Alphani Yapson, John Nolan, I Gede Eka Wiratnaya, I Gede Putu Supadmanaba

**Affiliations:** a Bachelor of Medicine and Medical Doctor Profession Study Program of Udayana University, Bali, Indonesia; b Department of Orthopedics and Traumatology, Faculty of Medicine, Udayana University-Prof IGNG Ngoerah General Hospital, Bali, Indonesia; c Department of Biochemistry, Faculty of Medicine, Udayana University, Bali, Indonesia

**Keywords:** Clinicopathology, CXCR4, Meta-analysis, Osteosarcoma, Prognosis

## Abstract

**Background:**

C-X-C Motif Chemokine Receptor (CXCR4) is an oncogene that widely studied and associated with worse clinicopathological features and prognosis outcome on many types of cancer. Beside that, significance of CXCR4 expression on clinicopathological features and prognostic on osteosarcoma (OS) require further validation.

**Aim:**

We conducted a meta-analysis to evaluate association between positive CXCR4 expression with clinicopathological features, and prognosis in OS.

**Methods:**

Literature searches on Pubmed, Cochrane Library and Web of Science was conducted systematically up to December 2021 to find relevant references. Effect of CXCR4 expression on clinicopathological characteristic and prognostic were analyzed using Review Manager 5.4 (Cochrane Collaboration, Oxford, UK). Significance value less than 0.05 was considered statistically significant.

**Results:**

By considering inclusion and exclusion criteria, 940 patients from 12 studies were suitable to included in qualitative analysis, and 10 studies were suitable for quantitative analysis. Association between CXCR4 expression and OS clinicopathological features was found significant on metastasis (OR = 4.01, 95%CI = 1.58–10.18; p = 0.003), stage (stage III & IV vs I & II, OR = 6.52, 95%CI = 1.05–40.62; p = 0.04), and tumor primary site (femur/tibia vs other, OR = 1.60, 95%CI = 1.04–2.45; p = 0.03), but not associated with histological type, gender, and age. Furthermore, CXCR4 expression is associated with poor overall survival in OS (HR = 2.13, 95%CI = 1.78–2.55; p < 0.001).

**Conclusion:**

In conclusion, the results of our meta-analysis suggest that CXCR4 expression may be valuable as a histopathological predictor of poor clinicopathological features and prognosis of OS.

## 1. Background

Osteosarcoma (OS) is the most common primary malignant bone tumor which is characterized by its rapid growth, strong invasiveness, and high rate of metastasis to the lungs, with a huge worldwide incidence [1]. It has a predilection for affecting children and adolescents between the ages of 10 and 19, predominantly occurs among males [2].

About 10–20% of osteosarcoma patients present with metastasis at early diagnosis, with the most common locations being the lung (85%), bone (8–10%), and lymph nodes. The presence of metastasis in patients with osteosarcoma develops poor prognosis and decreases survival rate outcomes [3,4]. Five-year overall survival for osteosarcoma patients is around 70% compared to patients with metastatic disease who show only 10%–30%. The current comprehensive treatment consisting of neoadjuvant chemotherapy and surgical resection has improved the five-year survival rate [5]. However, the prognosis in osteosarcoma with metastatic disease remains poor due to changes in chemotherapy regimen type, local recurrence, and type of metastasis leading to ineffective treatment [6,7]. Furthermore, 60% of osteosarcoma patients have not shown significant lung metastasis at initial diagnosis even though micrometastases exist [3,4]. Therefore, it is important to determine valuable prognostic markers for early detection of tumor metastasis and better understanding of clinicopathological changes that could improve the overall prognosis and survival of osteosarcoma patients significantly.

The C-X-C Motif Chemokine Receptor (CXCR4) is known as the most widely expressed chemokine receptor and is involved in numerous physiological and pathological conditions in the human body. It binds to its ligand named stromal derived factor-1 (SDF-1) that is expressed by most cells, including hematopoietic and endothelial cells in the lung, liver, skeletal muscle, and brain [8]. Colorectal, breast, kidney, lung, liver, and gallbladder cancers have all been associated with a poor prognosis when CXCR4 overexpression is present [9]. CXCR4 also contributes to angiogenesis, metastasis, tumor growth and invasion, even relapse and therapeutic resistance in some cancers [10]. Previous studies have shown a correlation of CXCR4 expression with prognosis in patients with bone and soft tissue sarcoma, but the uncertainty remains [11–21]. However, the prognostic role of CXCR4 expression in patients with osteosarcoma and its correlation with the clinicopathological features have not been analyzed specifically. Therefore, we performed this meta-analysis to identify studies from published literature and to evaluate whether expression of CXCR4 in patients with osteosarcoma can be a suitable prognostic and histopathological marker in the correlation to overall survival and clinicopathological features.

## 2. Method

We conducted searches in biomedical literature databases such as PubMed, Web of Science, and Cochrane Library. Implemented keywords were as follows: “Chemokine receptor type 4”, “C-X-C chemokine receptor type 4”, “CXCR4”, “osteosarcoma”, and “bone sarcoma”. The Boolean operators “AND” and “OR” are used to combine the keywords. The results were later filtered by considering inclusion criteria including: (1) written in English; (2) human research that included osteosarcoma patients that was confirmed by pathological or histological assessment; and (3) focused on exploration of correlation between CXCR4 positive expression with osteosarcoma prognostic and clinicopathological features. Studies were excluded if they met any of the following criteria: (1) non-English literature; (2) patents, cases, reviews, letters, and conference abstracts; and (3) non-human research. Then, two writers independently assessed the yielded literatures (GYAY and JN). Any disagreement on the eligibility of selected literatures were discussed to meet consensus. Study selection process is presented using the Preferred Reporting Items for Systematic Reviews and Meta-Analyses (PRISMA) flow chart [11]. The Newcastle Ottawa Scale (NOS) was used to assess the quality of the literature because all of the included studies were nonrandomized clinical trials. The NOS score 7–9 was considered high quality and was included in this review.

### 2.1. Data extraction

Demanded data were extracted, which include: (1) basic literature information (first author’s last name, year of publication, type of study, state/location of sample source, duration of follow-up, study period, and study design); (2) Patient sociodemographics, including race, mean age, gender, and CXCR4 expression status; (3) expression assessment method, which includes the assessment instrument, the source of the assessment instrument, the definition of positive expression/cut-off value, the expression site (nucleus, membrane, or cytoplasm), and the antibody type and dilution level if an immunohistochemistry (IHC) instrument is used; (4) clinicopathological outcome, including metastasis, tumor stage, primary tumor site, and histological type; (5) prognosis outcome, including overall survival data.

### 2.2. Statistical analysis

The role of positive or high CXCR4 expression on prognosis was calculated by pooled hazard ratio (HR), and on clinicopathological features (including metastasis, gender, age, tumor stage, histological type, and tumor site) were calculated by pooled odd ratio (OR). Hazard ratio data is used either from readily available data from literatures or extracted from another form of time-to-event data outcome with methods introduced by Tierney et al., both data are measured with a 95% confidence interval (95% CI) [12]. The meta-analysis model that used were determined by the analysis heterogeneity score (I^2^ statistic). The random effect model applied if I^2^ >50%; otherwise, fixed effect model is used. Publication bias was calculated by Begg’s funnel plot, indicated by its symmetry between logHRs or log-ORs, and its corresponding standard errors (SEs). Result with a significance value less than 0.05 (*p* < 0.05) was accounted as a statistically significant result. All statistical data analyses were conducted using Review Manager 5.4 (Cochrane Collaboration, Oxford, UK).

## 3. Result

### 3.1. Selected studies characteristics

**Fig. 1:**
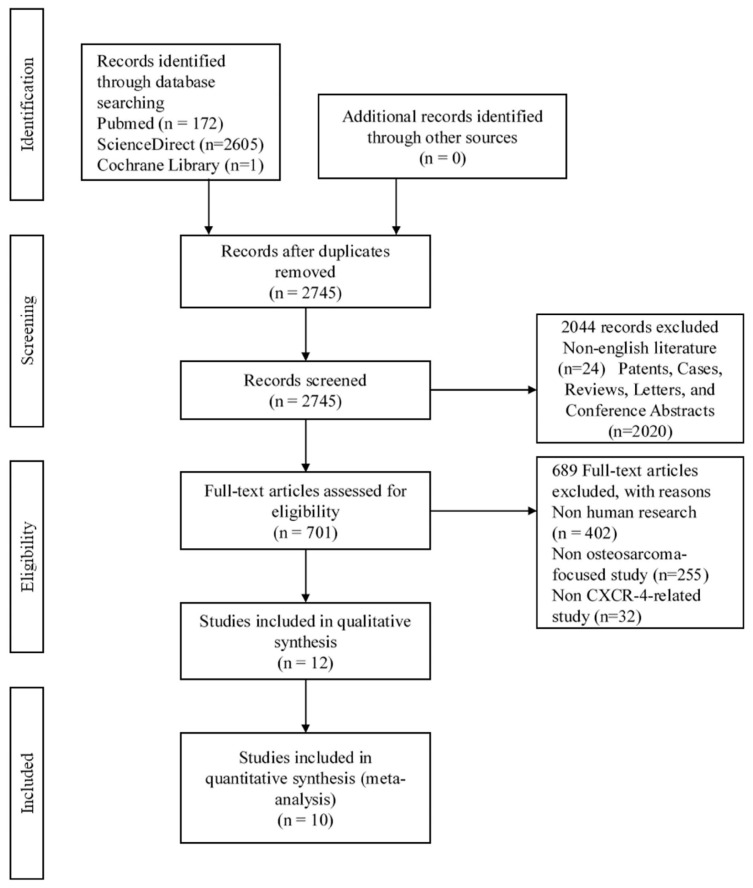
PRISMA Flow chart as visualization of study selection process.

Flow chart represents the selection process described in Fig. 1. Through the selection process, 12 studies were suitable to be included in qualitative analysis, and 10 studies were suitable to be included in quantitative analysis. General characteristics of 12 selected studies were extracted including study design, number of patients, sample source, age, and time to follow up duration, as shown in Table 1. In total, 940 OS patients were included as samples, mostly from Asian races (8/12 studies). NOS score for each study ranged from 7 to 9, an ideal score as mentioned in the method section.

In detail, quantitative measurement of CXCR4 methods of each study were also extracted. Immunohistochemistry (IHC) was mostly utilized in our selected eligible studies as a CXCR4 expression indicator, although the types, dilutions, and sources varied. Five studies also conducted reverse transcription polymerase chain reaction (RT-PCR) as a measurement instrument for CXCR4 expression. CXCR4 distribution assessed in each study ranged fromnucleus, cytoplasm, andmembrane, or a combination of those. Additionally, the definition of expression varied from each study; the immunostaining score, which ranged from 2–4, was frequently chosen as the cutoff number. Detailed characteristics of the included studies are shown in Table 2.

### 3.2. Association between CXCR4 expression and osteosarcoma clinicopathological features

Positive CXCR4 expression in OS and its correlation with metastasis was assessed in 10 studies with significant heterogeneity (I^2^ = 85%, *p* < 0.001). Pooled OR calculated under a random-effect model yielded 4.01 (95%CI = 1.58–10.18; *p* = 0.003), showed a significantly higher expression of CXCR4 in metastatic OS than in nonmetastatic OS.

CXCR4 expression in OS was not significantly associated with either male or female (OR = 0.83; 95%CI = 0.57–1.21; *p* = 0.33) and also unrelated to age (OR = 1.07; 95%CI = 0.71–1.62; *p* = 0.75). CXCR4 expression in OS was significantly associated with both features, which yielded pooled OR 6.52 (95%CI = 1.05–40.62; *p* = 0.04), under random-effect model, and 1.60 (95%Cl = 1.04–2.45, *p* = 0.03), under fixed-effect model, respectively. This means positive CXCR4 expression on OS was associated with higher tumor stages (III and IV), and more likely to appear in femur or tibia as its primary site.

**Fig. 2:**
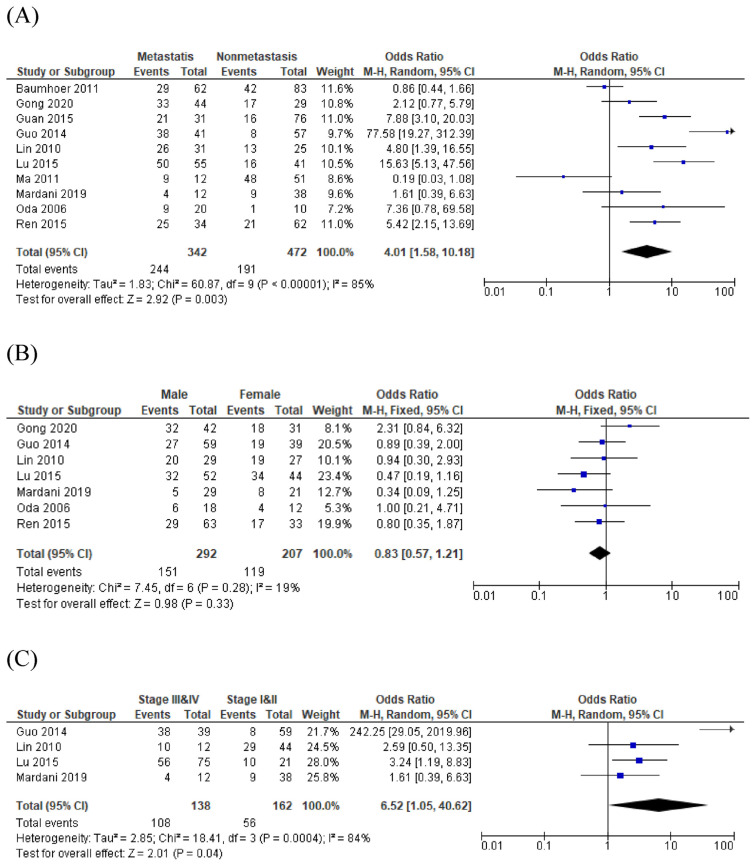

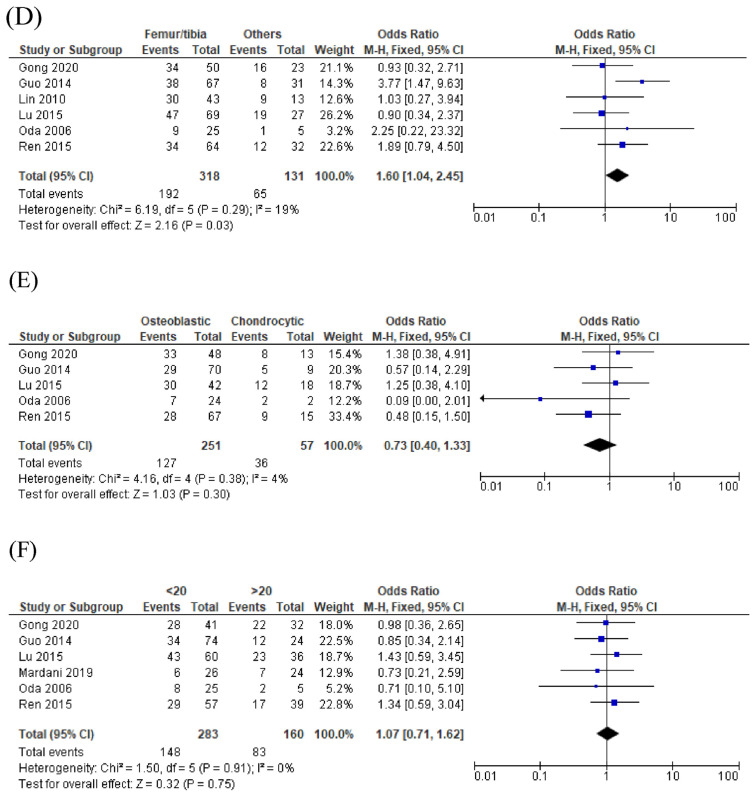
Forest plots show the association between CXCR4 positive expression and the following clinicopathological characteristics of OS: (A) metastasis, (B) gender, (C) stage, (D) and tumor site.

Considering histological type, CXCR4 expression in OS was not significantly associated with either osteoblastic or chondroblastic type, which yielded OR 0.73 (95%CI = 0.40–1.33, *p* = 0.30). Each analysis result was provided as a forest plot shown in Fig. 2.

### 3.3. Association between CXCR4 expression and osteosarcoma prognosis

**Fig. 3:**
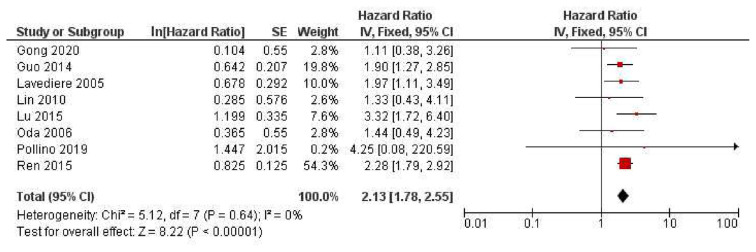
Forest plot represents the overall survival of patients with positive CXCR4 expression on OS.

CXCR4 expression and its effect on osteosarcoma patient’s overall survival was assessed in 8 studies that yielded a pooled HR 2.13 (95%Cl = 1.78–2.55, *p* < 0.001), meaning patients with high CXCR4 expression had a worse prognosis. The forest plot for survival analysis is shown in Fig. 3.

### 3.4. Sensitivity and publication bias

**Fig. 4:**
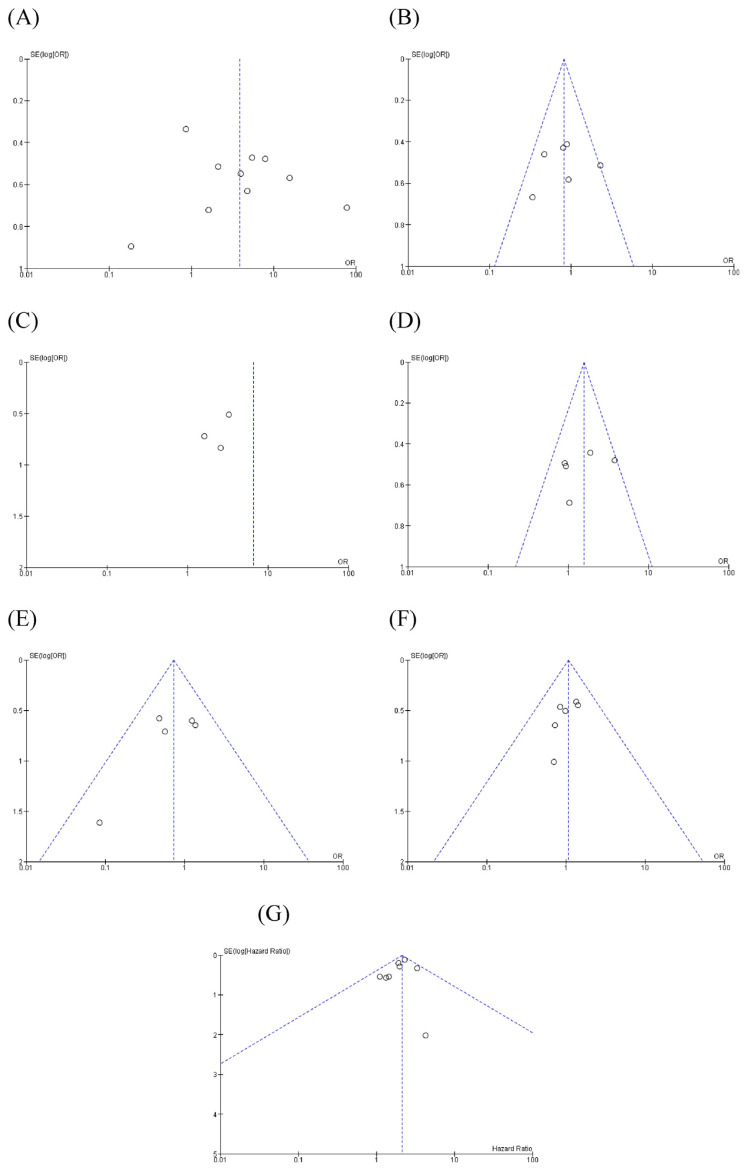
Begg’s funnel plot representing publication bias of each meta-analysis: (A) metastasis, (B) gender, (C) stage, (d) tumor site (e) histological type, (f) age and (g) hazard ratio. *SE(log[OR]) = Standard error multiplied log scale of odd ratio; SE(log[HR]) = Standard error multiplied log scale of hazard ratio.

Sensitivity test was done by removing studies one by one and assessing OR, HR, and 95%Cl value stability. The results are as follows: considering clinicopathology features, each feature had an OR ranged from 2.94 to 5.31 for metastasis, 0.7–0.94 for gender, 1.24–1.85 for tumor primary site, 0.61–0.86 for histological type, 0.99–1.13 for age, and HR ranged from 1.97 to 2.19 for overall survival. These findings showed consistent and stable results. The funnel plot of meta-analysis shows large symmetry, which suggests that no publication bias exists in the meta-analysis, as shown in Fig. 4.

## 4. Discussion

Osteosarcoma, the dominant primary bone malignancy that has high morbidity, and mortality rate, and may be acquired by all age groups, mostly in children and young adults [25]. Due to the high incidence in children and young adults, prolonging life expectancy by precision therapy is a necessary goal in the treatment of OS. Emerging evidence of prognostic factors contributes to early prevention of worsening conditions, complications, and decreased mortality risk. Gene-based prognostic factors also allow precise screening and suggest potential therapeutic targets for future personalized medicine, thus helping clinicians specify treatment options [26]. Recently, several specific biomarkers to determine prognostic factors have been found to formulate future therapy for OS [27].

Previous meta-analysis conducted by Li et al. [28], assessed the correlation of clinicopathological and prognostic significance of CXCR4 expression with soft tissue and bone sarcoma. The results were significant for tumor metastasis and stages. However, as the analysis merged patient’s criteria between different types of sarcomas, the results were unspecified for OS.

Current meta-analysis combined studies that assessed the association of CXCR4 expression with prognosis and clinicopathology characteristics specifically in OS patients. Along with previous reports, high expression of CXCR4 within the metastatic and advanced stage OS patients was also found. The possible mechanism behind this process is that CXCR4 plays a significant role in regulating the microtubule spindle. CXCR4 also held as an integral part of mitosis regulator and has an inhibitory effect on apoptosis [29,30]. CXCR4 expression in osteosarcoma cells showed that it enhances the proliferation of MG-63 cell lines and migration and inhibits apoptosis [27]. A recent study reported that the LM8 cell line found in mice with osteosarcoma expressed a high CXCR4 level. CXCR4 holds a major role in regulating the survival, migration, and apoptosis of LM8 cells [30]. The latest study reported that Mesenchymal Stem Cells (MSCs) within the tumor stroma and Vascular Endothelial Growth Factor (EFGR) advance the metastasis process [31]. The involvement of VEGF facilitates the metastasis of OS derived from MSCs, otherwise CXCR4 regulates the expression of VEGF of MSCs. Some studies reported that CXCR4 might modulate tumor cells to metastasize to a specific organ as it controls angiogenesis [32–35]. Some studies also found that the ability of CXCR4 to regulate neovasculature is accountable for addressing tumor cell metastasis, especially in solid organs [23,24,36].

The CXCR4 expression in OS based on gender separation showed no significant association for both male and female patients, the same result applied for age. Those findings were confirmatory, since they aligned with previous studies [23,27]. Inversely, CXCR4 expression significantly correlates with the tumor primary site. This finding is misaligned with previous studies may be due to the involvement of updated studies included in the analysis. When compared by its histological type, the meta-analysis shows no significant correlation either with the osteoclastic nor chondroblastic type. Currently, our meta-analysis shows up to be the first analysis that assesses the correlation of CXCR4 expression with OS histological type.

The majority of previous studies showed a diminished level of survival possibility which aligned with our result. The group with CXCR4 expression also showed a depleted survival rate in other bony malignancies [37]. This poor prognosis may correlate with the metastatic process, and tumor stage that significantly higher CXCR4 expression [27]. Furthermore, the findings of those CXCR4 expression primary site in OS patients might help physicians to determine the strategy toward the disease. Physicians should be more aware of the worsening clinicopathological features and prognosis in such cases. Further exploration of therapy focusing on CXCR4 may also be developed.

Regarding publication bias, we performed Begg’s funnel plot test in order to assess any publication bias. The funnel plot of the meta-analysis shows large symmetry which suggests that no publication bias exists in the meta-analysis. Sensitivity tests were also performed, which yielded consistent and stable results. Several limitations of this study should also be noted that this study’s inclusion criteria were limited to only English literature and may be biased by inaccessible data in some excluded studies. Another limitation of this study was determining the HR, as some papers may not provide it directly. By then, we need to calculate directly from the Kaplan–Meier curves in the papers. Further studies should be conducted in order to confirm this interpretation.

To conclude, this study demonstrated CXCR4 is significantly related to metastasis, tumor stage, tumor primary site, and overall survival in OS. Possible advantages provided by this study might be to consider CXCR4 expression as either a prognostic factor or novel therapeutic target. Furthermore, to confirm our findings, additional well-designed research is necessary.

## Figures and Tables

**Table 1:** General characteristics of each study selected for the meta-analysis.

No.	Study	Study design	Sample source	Number of patients	Age	Time of follow up (months)	NOS Score
**1**	Gong, 2020 [13]	Prospective	China	73	NA	36	9
**2**	Mardani, 2019 [14]	Prospective	Iran	50	19 ± 8.9	NA	7
**3**	Pollino, 2019 [15]	Prospective	Italy	48	19	48	9
**4**	Ren, 2015 [16]	Retrospective	China	96	NA	57.5 (6–171)	8
**5**	Lu, 2015 [17]	Retrospective	China	96	18 (8–49)	Minimum 36	9
**6**	Guan, 2015 [18]	Prospective	China	107	16.5 (8–48)	34 (5–65)	8
**7**	Guo, 2014 [19]	Retrospective	China	98	NA	NA	7
**8**	Ma, 2011 [20]	Retrospective	China	63	16 (8–48)	66 (12–120)	8
**9**	Baumhoer, 2011 [21]	Retrospective	Germany	145	22.9 (4–88)	NA	8
**10**	Lin, 2010 [22]	Retrospective	China	56	22.4 (7–67)	33.5 (20–75)	7
**11**	Oda, 2006 [23]	Retrospective	Japan	30	15 (7–69)	38.5 (9–145)	8
**12**	Laverdiere, 2005 [24]	Retrospective	USA	47	16 (4–77)	41 (1–329)	7

NA: Not Applicable.

**Table 2:** Detailed characteristic of each study selected for the meta-analysis.

No	Study	Method(s)	Antibody Source for IHC	Antibody type	Antibody dilution	Definition of CXCR4 expression	CXCR4 cellular distribution
**1**	Gong,2020	IHC	Santa cruz Biotechnology	Polyclonal	1:100	Total IHC score ≥4	Cytoplasm
**2**	Mardani, 2019	IHC	Medaysis RM0407RTU7	Monoclonal	NA	Total IHC Score ≥3	Cytoplasm and membrane
**3**	Pollino,2019	RT-PCR, IHC	Thermo Fisher Scientific, Abcam ab2074.	Monoclonal	1:1000	Total IHC Score ≥3	Cytoplasm and nucleus
**4**	Ren,2015	IHC	Abcam ab2074	Polyclonal	1:100	Total IHC score ≥4	Cytoplasm
**5**	Lu,2015	qRT-PCR IHC	Invitrogen Life Technologies, Abcam ab2074	Polyclonal	NA	Total IHC score ≥4	Cytoplasm
**6**	Guan,2015	qRT-PCR, IHC	Roche Molecular Biochemicals, R&D System	Monoclonal	1:75	Total IHC score ≥5	NA
**7**	Guo,2014	qRT-PCR, IHC	ab58176, Abcam	Monoclonal	1:200	Total IHC Score ≥2	Cytoplasm and membrane
**8**	Ma, 2011	IHC	Santa Cruz Biotechnology	Monoclonal	1:100	NA	Nucleus, cytoplasm and membrane
**9**	Baumhoer, 2011	IHC	Ventana BenchMark XT	Polyclonal	1:500	Total IHC Score ≥3	Cytoplasmic and membrane
**10**	Lin,2010	IHC	Boster Biological Technology,	Polyclonal	1:400	Nuclear or cytoplasmic staining Positive cells >1%	Cytoplasm and nucleus
**11**	Oda,2006	IHC	BD Pharmingen clone 125G	Monoclonal	1:100	Total IHC score ≥2	Cytoplasm and nucleus
**12**	Laverdiere, 2005	qRT-PCR	NA	NA	NA	NA	NA

IHC: Immunohistochemistry; RT-PCR: reverse transcription polymerase chain reaction.
